# A-Kinase Anchoring Protein 4 (AKAP4) is an ERK1/2 substrate and a switch molecule between cAMP/PKA and PKC/ERK1/2 in human spermatozoa

**DOI:** 10.1038/srep37922

**Published:** 2016-11-30

**Authors:** Liat Rahamim Ben-Navi, Tal Almog, Zhong Yao, Rony Seger, Zvi Naor

**Affiliations:** 1 Department of Biochemistry and Molecular Biology, The George S. Wise Faculty of Life Sciences, Tel Aviv University, Ramat Aviv 69978, Israel; 2 Department of Biological Regulation, the Weizmann Institute of Science Rehovot 76100, Israel

## Abstract

Mammalian spermatozoa undergo capacitation and acrosome reaction in order to fertilize the egg. The PKC-ERK1/2 pathway plays an important role in human spermatozoa motility, capacitation and the acrosome reaction. Here we demonstrate that ERK1/2 phosphorylates proAKAP4 on Thr265 in human spermatozoa *in vitro and in vivo*. Cyclic AMP (cAMP) had no effect on ERK1/2 activity in human spermatozoa, but stimulated the MAPK in mouse pituitary LβT2 gonadotrope cells. cAMP via PKA attenuates PKC-dependent ERK1/2 activation only in the presence of proAKAP4. St-HT31, which disrupts PKA-regulatory subunit II (PKA-RII) binding to AKAP abrogates the inhibitory effect of cAMP in human spermatozoa and in HEK293T cells expressing proAKAP4. In transfected HEK293T cells, PMA relocated proAKAP4, but not proAKAP4-T265A to the Golgi in an ERK1/2-dependnet manner. Similarly, AKAP4 is localized to the spermatozoa principal piece and is relocated to the mid-piece and the postacrosomal region by PMA. Furthermore, using capacitated sperm we found that cAMP reduced PMA-induced ERK1/2 activation and acrosome reaction. Thus, the physiological role of the negative crosstalk between the cAMP/PKA/AKAP4 and the PKC/ERK1/2 pathways is to regulate capacitation and acrosome reaction.

Mammalian spermatozoa acquire the ability to fertilize the egg during their path to the oocyte. Spermatozoa undergo a series of biochemical and physiological processes known as capacitation^1^ (For Review). Capacitated sperm exhibit forward motility and in addition a unique type of motility, hyperactivation. In addition, capacitation is associated with reorganization of the plasma membrane, cholesterol efflux, PKA-dependent protein tyrosine phosphorylation, intracellular alkalization, increase in intracellular Ca^2+^ concentration, and increase in cAMP. Capacitation ends in the process of acrosome reaction and fertilization of the egg^2^,^3^,^4^,^5^,^6^,^7^,^8^,^9^,^10^. The occurrence of sperm capacitation is a prerequisite for a normal acrosome reaction and fertilization.

A-kinase anchoring proteins (AKAPs) are a family of scaffolding proteins that target PKA and other signaling enzymes to selective subcellular locations^11^. The AKAP family includes more than 50 structurally diverse but functionally similar members that contain at least one PKA anchoring domain. AKAPs have unique localization signals and the ability to form complexes with other signaling molecules. Hence, AKAPs may serve as platforms for the integration of cAMP and other signaling pathways as they can bind other protein kinases, protein phosphatases, ion channels, and small GTP binding proteins^12^,^13^,^14^,^15^.

Evidence for the involvements of AKAPs in the PKC-ERK1/2 pathway was presented by Su *et al*.^16^, who found that SSeCKS/Gravin/AKAP12 inhibits cancer invasiveness and chemotaxis by suppression of the PKC-Raf/MEK/ERK1/2 pathway. In contrast, AKAP13/AKAP-LBC binds KSR-1 and facilitates the activation of ERK1/2 following cAMP treatment^17^. Thus, the crosstalk between AKAPs and the PKC-ERK1/2 pathways is cell context-dependent.

AKAP4 (also named AKAP82) is a one of the major components (~50%) of the sperm fibrous sheath, a structure known to be involved in sperm motility. AKAP4 has a precursor termed proAKAP4 that is processed to mature AKAP4 during sperm differentiation in the human testes. Both proteins are highly homologous to mouse AKAP4. In contrast to mouse AKAP4, there is still a considerable portion of proAKAP4 that is not processed to AKAP4, and is localized to the entire sperm principal piece^18^. AKAP4 has two binding sites for PKA, one is a dual specificity site and binds RIα or RIIα subunits of PKA, and another site that binds only the RIα subunit^19^. Binding sites for PKC are polybasic amino acid residues, namely they contain multiple lysines and/or arginines^20^,^21^. AKAP4 has several putative binding sites for PKC. In addition, AKAP4 and proAKAP4 were found to be serine- and tyrosine-phosphorylated in a capacitation-dependent manner in human spermatozoa but the nature of the kinases involved were not yet elucidated^22^. Spermatozoa from mice lacking AKAP4 failed to show progressive motility and the male mice were infertile^23^.

We and others have previously reported that the PKC/ERK1/2 pathway plays an important role in human sperm motility, capacitation and acrosome reaction^24^,^25^,^26^,^27^. Here we demonstrate for the first time that AKAP4 is an ERK1/2 substrate and a regulator of the cAMP/PKA and the PKC/ERK1/2 pathways in human spermatozoa. Furthermore, we show that the physiological role of the negative crosstalk between the cAMP/PKA/AKAP4 and the PKC/ERK1/2 pathways is to regulate capacitation and acrosome reaction.

## Results

### AKAP4 is an ERK1/2 substrate in human spermatozoa

**Figure 1:**
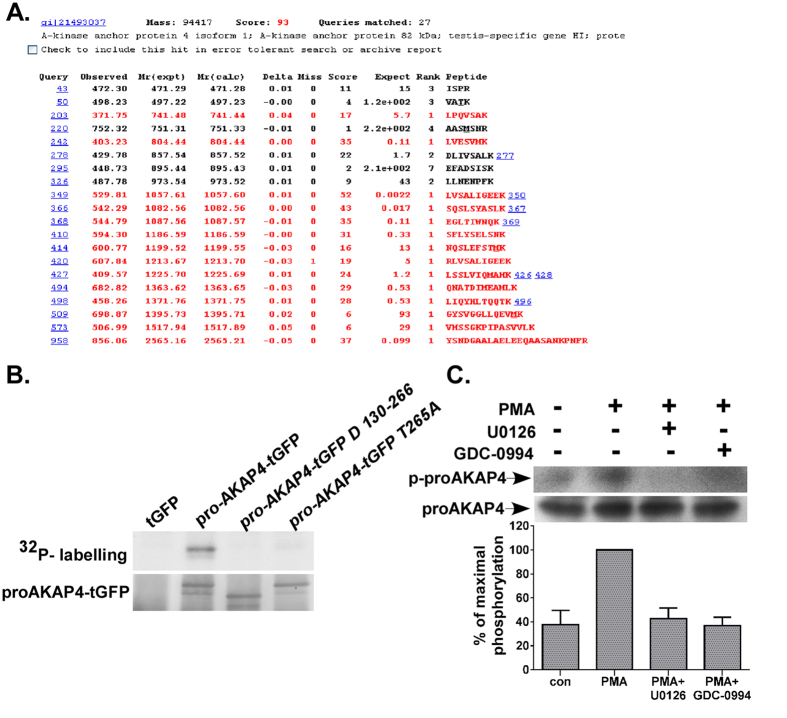
AKAP4 is an ERK1/2 substrate. (**A**) AKAP4 is a potential MAPK substrate. Human spermatozoa were stimulated with 100 nM PMA for 15 min and subjected to western-blot with anti-MPM2, a phospho-specific anti-MAPK substrate antibody. A major PMA-stimulated band (~90 kDa) was subjected to MALDI-TOF mass spectrometry. The peptide coverage map of the specific identified protein indicated that the protein is AKAP4 (82 kDa). (**B**) proAKAP4 is phosphorylated *in vitro* by ERK2 on Thr265. HEK293T cells were transfected with proAKAP4 fused to turboGFP in its N-terminus using the calcium phosphate method. Cells were also transfected with turboGFP, turboGFP-proAKAP4^130–266^ deletion mutant and turboGFP-proAKAP4^T265A^ mutant. Later cells were lysed, and the substrates were immunoprecipitated with an anti-turboGFP antibody. The precipitates were subjected to an *in vitro* phosphorylation assay with activated ERK2 as mentioned under experimental procedures. The upper panel is the autoradiogram, and the lower panel is a western blot of anti-tGFP. Lanes from left to right are as followes: tGFP, tGFP-proAKAP4, tGFP-proAKAP4 delta (130–266), and tGFP-proAKAP4-T265A. The experiment is a representative result from three experiments. (**C**) proAKAP4 is phosphorylated by ERK1/2 in spermatozoa *in vivo*. Human spermatozoa were washed and pretreated with U0126 (25 μM) or GDC-0994 (10 μM) for 20 min. Thereafter, the cells were treated with or without PMA (25 nM) for 15 min. Cells lysate were immunoprecipitated with anti-proAKAP4, followed by western blotting with anti-MPM2, or anti-proAKAP4 for loading control. The experiment is a representative result.

We have previously reported that the PKC-ERK1/2 pathway plays an important role in human sperm functions^26^. These findings motivated the search for spermatozoa ERK1/2 substrates. To that end, we took a proteomic approach, using anti-MPM2 antibodies that recognize MAPK phosphorylation motifs, namely proline-directed Ser/Thr sequences (P-S/T), to identify MAPK hyper-phosphorylated substrates. Several hyper-phosphorylated bands ranging from 60–100 kDa were identified following PMA treatment of human spermatozoa^26^. Bands from the corresponding molecular weight of 60–100 kDa were cut from the gel and subjected to MALDI-TOF mass spectrometry. The search revealed two MAPK substrates, the first was ARHGAP6^26^ and the second was AKAP4 (Fig. 1A). AKAP4 and its precursor, proAKAP4, are major components of the human spermatozoa fibrous sheath, a vital regulator of spermatozoa motility^18^. ProAKAP4 and AKAP4 contain several potential MAPK phosphorylation sites. The potential residues in proAKAP4 that can be phosphorylated by MAPK are Ser130, Ser190, Ser207, Ser254 and Ser262, which have the minimal MAPK phosphorylation sequence, Ser-Pro or Thr-Pro. In addition, proAKAP4 contains a PRTP sequence (residues 263–266) that is a classical MAPK phosphorylation motif and hence Thr265 is a classical potential phosphorylation site. We then tested whether proAKAP4 is phosphorylated by ERK2 on Thr265 *in vitro* (Fig. 1B). We used the following substrates: turboGFP, turboGFP-proAKAP4, turboGFP-proAKAP4^130–266^ deletion mutant and turboGFP-proAKAP4^T265A^ mutant. The proteins were overexpressed in HEK293T cells, immunoprecipitated with anti-turbo-GFP and subjected to *in vitro* phosphorylation assay by ERK2. Indeed, ERK2 phosphorylated proAKAP4 *in vitro* (Fig. 1B). The proAKAP4^130–266^ deletion mutant (Δ) lacking all the potential MAPK phosphorylation sites was not phosphorylated and the proAKAP4^T265A^ mutant exhibited a faint phosphorylation band that was hardly detected (Fig. 1B). The data indicate that proAKAP4 is phosphorylated *in vitro* by ERK2 and that the major phosphorylation site is Thr265. We then examined the phosphorylation of proAKAP4 by MAPK *in vivo*. Spermatozoa were incubated with PMA, lysed and immunoprecipitated with proAKAP4 antibodies followed by western blotting with anti-MPM2 antibodies. Indeed, proAKAP4 was phosphorylated *in vivo* by MAPK after incubation with PMA for 15 min (Fig. 1C). Preincubation with the MEK inhibitor, U0126, or the ERK1/2 inhibitor, GDC-0994 which prevents ERK1/2 activity through steric effects, without preventing MEK phosphorylation of ERK1/2, abolished the PMA-induced phosphorylation of proAKAP4 by MAPK (Fig. 1C), indicating that the MAPK involved is ERK1/2.

### Activation of PKA by cAMP inhibits PKC-dependent activation of ERK1/2 in human spermatozoa

**Figure 2:**
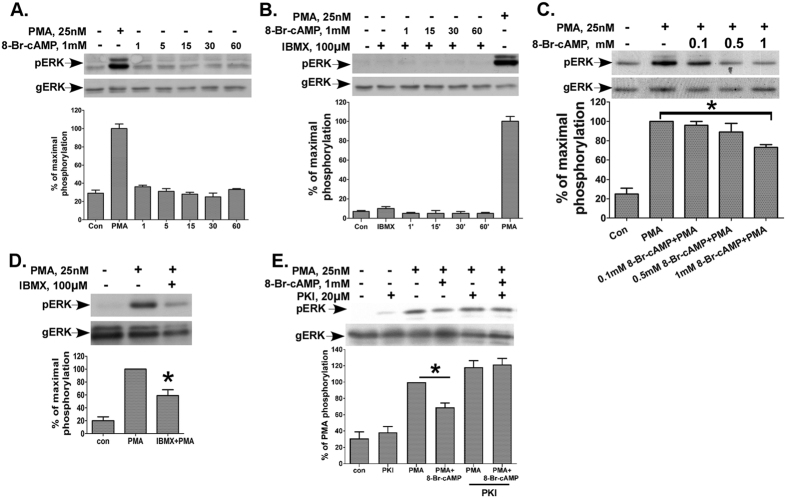
cAMP via PKA inhibits PKC-dependent activation of ERK1/2 in human spermatozoa. (**A**) cAMP has no effect on ERK1/2 activation in human spermatozoa. Cells were incubated with 1 mM 8-Br-cAMP for the indicated times, while PMA (25 nM, 15 min) served as a positive control. Cells were then lysed and analyzed for ERK2 activity by western blotting using an antibody for phospho-ERK1/2 (pERK). Total ERK1/2 (gERK) was detected with polyclonal antibodies as a control for sample loading. In this and subsequent figures representative blots are shown and *bars* are mean ± SEM from three experiments. (**B**) cAMP and IBMX have no effect on ERK2 activity in human sperm. Human spermatozoa were preincubated with or without 100 μM IBMX for 30 min. 8-Br-cAMP (1 mM) was then added for the indicated times (in minutes) and ERK2 activity was determined as above. (**C**) cAMP inhibits PMA-induced ERK1/2 activation in human spermatozoa. Human spermatozoa were incubated with or without 0.1 mM, 0.5 mM or 1 mM 8-Br-cAMP for 10 min followed by 25 nM PMA for additional 15 minutes and ERK2 activity was determined as above. (**D**) IBMX inhibits PMA-induced ERK1/2 activation in human spermatozoa. Human spermatozoa were preincubated with or without 100 μM IBMX for 30 minutes and PMA (25 nM) was then added for 15 min and ERK2 activity was determined as above. (**E**) PKA mediates the cAMP inhibition of PMA-induced ERK2 activation in human spermatozoa. Human spermatozoa were washed and preincubated with the PKA inhibitor PKI (20 μM) for 1 h, with or without 8-Br-cAMP (1 mM) for 10 min. Thereafter PMA (25 nM) was added for another 15 min. Cell lysates were analyzed for ERK2 activity by Western blotting as above. A representative blot is shown and *bars* are mean ± SEM. from three experiments. Means designated by * are significantly different (p < 0.05).

Since AKAP4 is a PKA anchor protein and is an ERK1/2 substrate; we decided to examine the crosstalk between the cAMP/PKA/AKAP4 and the PKC/ERK1/2 pathways, both known to play a major role in sperm biology. cAMP is an important regulator of sperm motility^28^,^29^, capacitation^3^ and the acrosome reaction^30^. We and others have shown that the PKC/ERK1/2 pathway is involved in sperm motility, capacitation and acrosome reaction^24^,^25^,^26^,^27^. Since cAMP exerts opposite effects on the ERK1/2 pathway in different cells^31^,^32^, it was interesting to examine the effect of cAMP on ERK1/2 activation in human spermatozoa. Addition of 8-Br-cAMP, a cell-permeable analog of cAMP, to human spermatozoa had no effect on ERK1/2 activation, while a positive control with PMA is shown (Fig. 2A). To rule out that the lack of effect was due to increased phoshpodiesterase (PDE) activity, we pre-incubated the cells with IBMX, a phoshpodiesterase inhibitor, followed by 8-Br-cAMP treatment and still could not find a change in ERK1/2 activity (Fig. 2B). We then postulated that cAMP may negatively crosstalk with the PKC/ERK1/2 pathway in human spermatozoa. Therefore, 8-Br-cAMP was added to noncapacitated sperm 10 min before PMA. Indeed, 8-Br-cAMP decreased ERK1/2 activation by PMA in a dose dependent fashion (Fig. 2C). Similarly, IBMX reduced the PMA-activation of ERK1/2 (Fig. 2D). We then examined whether the inhibitory effect of cAMP on ERK1/2 activation by PMA is PKA-dependent. Incubation of human spermatozoa with the PKA inhibitor PKI had a slight stimulatory effect upon basal ERK1/2 activity, which was not significant (Fig. 2E). As before, cAMP reduced ERK1/2 activation by PMA. However, the PKA inhibitor, PKI abolished the inhibitory effect of 8-Br-cAMP on PMA induced ERK1/2 activation. Hence, the cAMP inhibition of PMA-activation of ERK1/2 in human spermatozoa is mediated by PKA.

### cAMP stimulates ERK1/2 activity in mouse pituitary LβT2 gonadotrope cells

**Figure 3:**
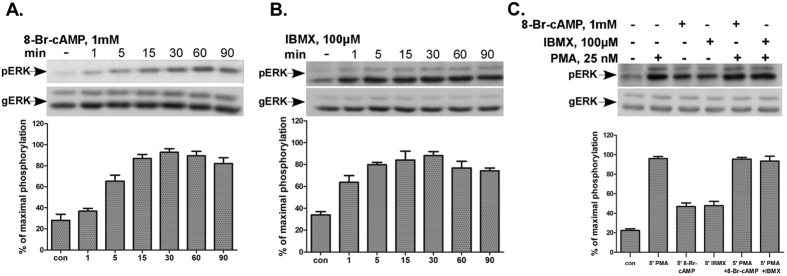
cAMP stimulates ERK1/2 activity in mouse pituitary LβT2 gonadotrope cells. (**A,B**) cAMP and IBMX stimulate ERK2 activation in mouse pituitary LβT2 gonadotrope cells. Cells were serum starved for 16 h before treatment with 1 mM 8-Br-cAMP (**A**) or 100 μM IBMX (**B**) for the indicated times. ERK2 activity was determined as above. (**C**) cAMP and IBMX do not inhibit PMA-stimulation of ERK1/2 activation in LβT2 cells. Cells were serum starved for 16 h before pretreatment with 1 mM 8-Br-cAMP, or IBMX (100 μM) for 10 and 30 min respectively. Thereafter PMA (25 nM) was added for 5 min and ERK2 activity was determined as above. Representative blots are shown and bars are mean ± SEM from three experiments.

To verify whether the lack of effect by cAMP on basal ERK1/2 activity and the inhibitory effect upon PMA-induced ERK1/2 activity is cell-specific, we examined the effect of cAMP on ERK1/2 phosphorylation in LβT2 gonadotrope cells^33^, in which a robust activation of ERK1/2 by GnRH has been observed^34^,^35^. 8-Br-cAMP and IBMX stimulated an increase in ERK1/2 activation with a peak at 30 min, while the effect lasted for at least 90 min (Fig. 3A,B). We then examined whether cAMP attenuates the activation of PKC/ERK1/2 pathway as we had seen in human spermatozoa. Therefore 8-Br-cAMP or IBMX were added to the cells 10 and 30 min, respectively, before stimulation with PMA for 5 min (in LβT2 cells and spermatozoa, PMA exerts a maximum effect on ERK1/2 activation at 5 and 15 min, respectively)^26^,^35^,^36^. Unlike the effect observed in sperm, 8-Br-cAMP and IBMX did not attenuate the activation of ERK1/2 by PMA (Fig. 3C). We conclude that cAMP regulation of ERK1/2 activity is cell context-dependent.

### cAMP inhibits PMA-induced ERK1/2 activation in HEK293T cells expressing proAKAP4

**Figure 4:**
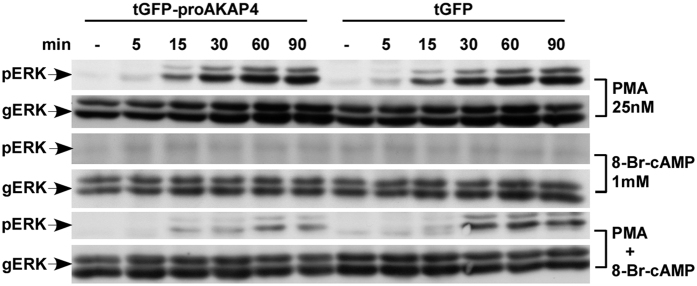
cAMP inhibits PMA-induced ERK1/2 activation in HEK293T cells expressing proAKAP4. HEK293T cells were transfected with turboGFP-proAKAP4 or with turboGFP. Following the transfection (48 hours) the cells were preincubated with or without 8-Br-cAMP (1 mM) for 10 min, followed by treatment with or without PMA (25 nM) for the indicated time (0–90 min). After treatment, cell lysates were analyzed for ERK1/2 activity as in Fig. 2. A representative blot is shown from three similar experiments. 8-Br-cAMP (lanes 5 and 6), had a pronounced inhibitory effect on PMA-stimulated ERK1/2 activation in the tGFP-proAKAP4 vs. the tGFP expressing cells.

The above results led us to hypothesize that AKAP4 can play a role in regulating spermatozoa cAMP/PKA and the PKC/ERK1/2 pathways, both known to regulate sperm biology^3^,^24^,^25^,^26^,^27^,^28^,^29^,^30^,^37^,^38^,^39^. We therefore examined the effect of cAMP upon PMA-stimulated ERK1/2 activity in the presence or absence of proAKAP4 in HEK293T cells. Unlike spermatozoa, which are fully differentiated and cannot be transfected, HEK293T cells can be readily transfected and we took advantage of this feature in order to test our hypothesis. HEK293T cells were transfected with turboGFP-proAKAP4, or turboGFP alone, which served as a control, and then treated with PMA, with or without 8-Br-cAMP for 5–90 min (Fig. 4). PMA treatment resulted in a robust ERK1/2 activation, with no difference between tGFP-proAKAP4 and tGFP (upper 2 lanes). On the other hand, 8-Br-cAMP did not stimulate ERK1/2 activation in the two groups of cells, similar to what we saw in spermatozoa (lanes 3 and 4). However, 8-Br-cAMP (lanes 5 and 6), had a pronounced inhibitory effect on PMA-stimulated ERK1/2 activation only in the tGFP-proAKAP4 expressing cells. The data suggest that proAKAP4 is involved in the cAMP inhibition of ERK1/2 activation by PMA in HEK293T cells and possibly also in spermatozoa.

### Association between Raf-1 and proAKAP4

**Figure 5:**
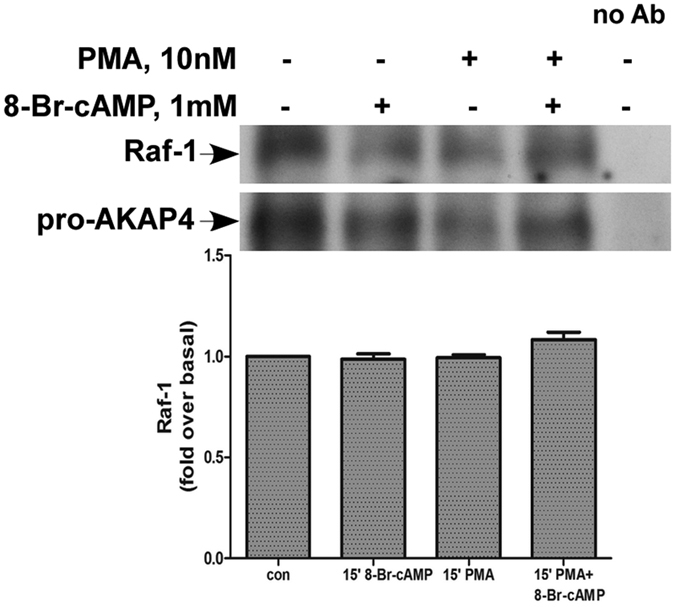
Association between Raf-1 and proAKAP4. Human spermatozoa were washed and preincubated with or without 8-Br-cAMP(1 mM) for 10 min followed by treatment with or without PMA (25 nM) for 15 min. Cells lysate were immunoprecipitated with anti-AKAP4, followed by western blotting with anti-Raf-1 or anti-AKAP4 for loading control. Beads without anti-AKAP4 antibody were incubated with cell lysates to serve as antibody specificity control (lane 5). A representative blot is shown and *bars* are mean ± SEM from three similar experiments. Although a decrease is observed in lanes 2, 3, note that a similar decrease is observed in the amount of the immunoprecipitated proAKAP4.

The ERK1/2 pathway consists of A/B/C-Raf-MEK1/2 and ERK1/2. Human spermatozoa are known to express the Raf-1 (C-Raf), but not the A and B-Raf^40^ and mainly ERK2^26^. Also, ERK1/2, Raf-1 and AKAP4 have been localized to human spermatozoa tail^26^,^40^. In order to identify how proAKAP4 regulates the cAMP/PKA and the PKC/ERK1/2 pathways we tested whether it associates with Raf-1 (Fig. 5). Spermatozoa were incubated with PMA, 8-Br-cAMP or with PMA and 8-Br-cAMP for 15 min, followed by immunoprecipitation of proAKAP4 and western blotting with anti Raf-1 antibody (Fig. 5). The data shows that proAKAP4 interacts with Raf-1 under basal and activated conditions, supporting the notion that proAKAP4 can bridge between Raf-1-MEK1/2-ERK1/2 and the cAMP/PKA pathways.

### St-HT31 abolished the cAMP inhibition of PMA-induced ERK1/2 activation in human spermatozoa

**Figure 6:**
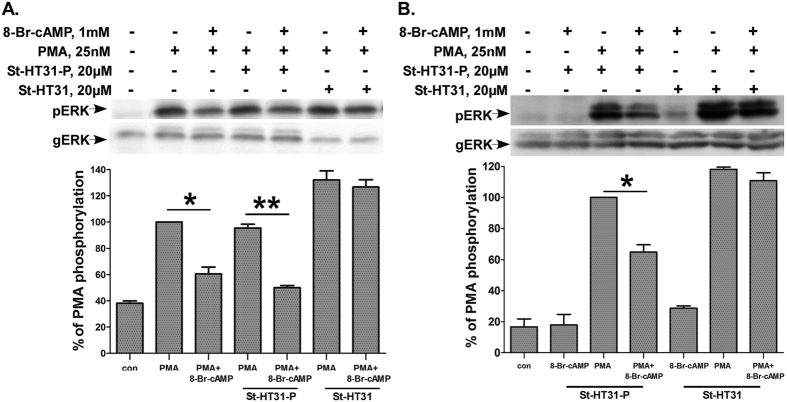
St-HT31 abolished the cAMP inhibition of PMA-induced ERK1/2 activation. (**A**) St-HT31 abolished the cAMP inhibition of PMA-induced ERK2 activation in human spermatozoa. Human spermatozoa were washed and preincubated with St-HT31 or the inactive peptide inhibitor St-HT31-P (both 20 μM) for 20 min followed by another preincubation with or without 8-Br-cAMP (1 mM) for 10 min. Thereafter PMA (25 nM) was added for another 15 min. cell lysates were analyzed for ERK2 activity by western blotting as above. A representative blot is shown and *bars* are mean ± SEM from three experiments. (**B**) St-HT31 abolished the cAMP inhibition of PMA-induced ERK1/2 activation in HEK293T cells expressing proAKAP4. HEK293T cells were transfected with turboGFP-proAKAP4. Following the transfection (48 hours) cells were pretreated with St-HT31, or St-HT31-P (20 μM) for 20 min, and were then preincubated with or without 8-Br-cAMP (1 mM) for 10 min. Thereafter PMA (25 nM) was added for another 60 min. Cell lysates were analyzed for ERK2 activity by Western blotting as above. A representative blot is shown and *bars* are mean ± SEM. from three experiments. *(p < 0.05) **(p < 0.01).

We then investigated whether the inhibitory effect of cAMP on the PKC/ERK1/2 pathway is AKAP-dependent. Disruption of PKARII anchoring to AKAP can be achieved by using St-HT31, an anchoring inhibitor peptide derived from the RII binding domain of a human thyroid AKAP^41^. Stearated HT- 31 (St-HT31) is a cell-permeable version of this peptide that was shown to inhibit sperm motility^42^. We therefore examined the effect of St-HT31 on cAMP inhibition of PMA-induced ERK1/2 activation. Spermatozoa were pre incubated with St-HT31 or St-HT31-P (a non-active control peptide) for 20 min and PMA or PMA and 8-Br-cAMP were added. St-HT31 abolished the inhibitory effect of 8-Br-cAMP on PMA induced ERK1/2 activation while St-HT31-P had no effect (Fig. 6A). Hence, AKAP is involved in cAMP inhibition of PMA to ERK1/2 signaling in human spermatozoa.

### St-HT31 abolished the cAMP inhibition of PMA-induced ERK1/2 activation in HEK293T cells expressing proAKAP4

In order to specifically assess the involvement of proAKAP4 in the inhibitory effect of cAMP on the PKC-ERK1/2 pathway we repeated the experiment in HEK293T cells expressing proAKAP4 (Fig. 6B). Indeed, St-HT31 abolished the inhibitory effect of 8-Br-cAMP on PMA induced ERK1/2 activation, while St-HT31-P had no effect. The data supports the view that AKAP4 is involved in cAMP inhibition of PMA-induced ERK1/2 activation in HEK293T and in human spermatozoa.

### proAKAP4 translocates to the Golgi in HEK293T cells following PMA treatment

**Figure 7:**
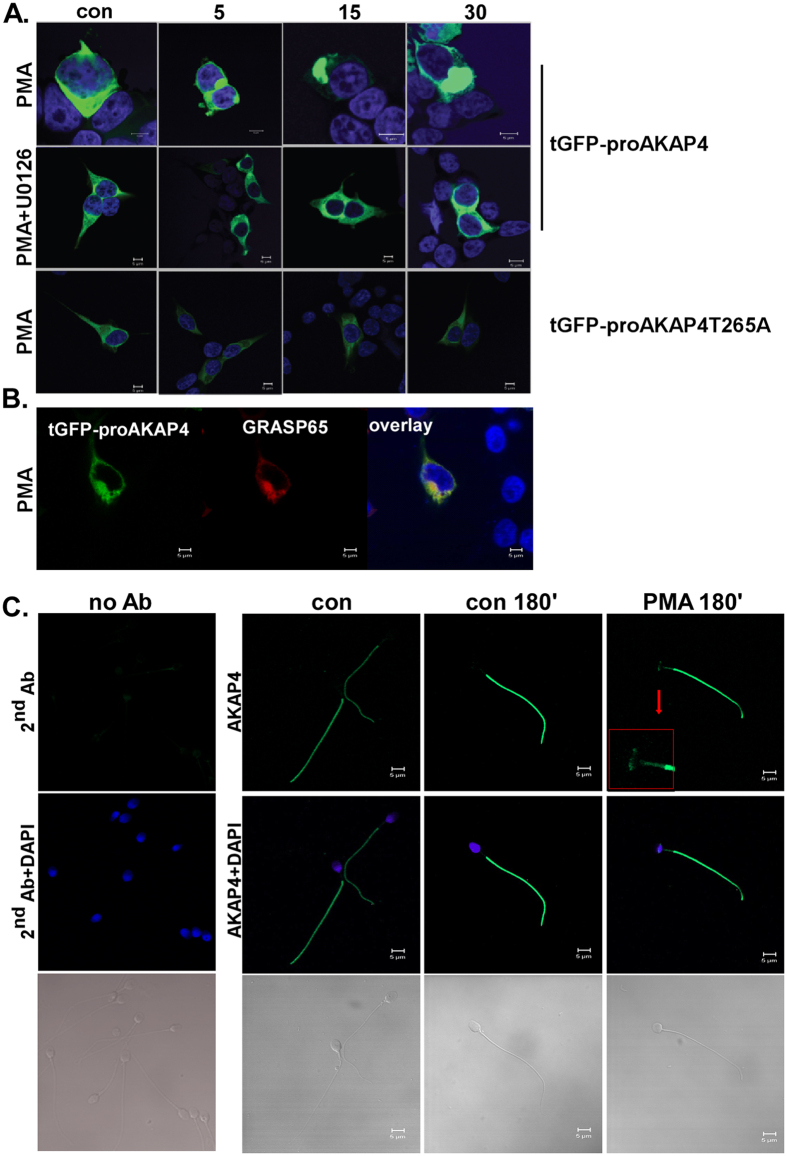
Localization of AKAP4. (**A,B**) Fate of AKAP4-tGFP and AKAP4-T265A in HEK293T. HEK293T were transfected with tGFP-proAKAP4 or with tGFP-proAKAP4-T265A. Serum-starved cells, 48 h after transfection, were pretreated with or without U0126 (10 μM) for 20 min. Thereafter PMA (25 nM) was added for different time points. (**B**) Golgi localization was visualized by cotransfection with GRASP65-RFP. Formalin-fixed slides were imaged under a 63 objective on Zeiss confocal microscope. At least 10 images from each treatment were collected and a representative image is shown. The *scale bar* is 5 μm. (**C**) Fluorescence microscopy for expression of AKAP4 in human spermatozoa. Human spermatozoa were treated with PMA (25 nM) for 180 min and then were reacted with anti-AKAP4 antibody and DAPI. Negative control with secondary antibody is shown on the left. Note that AKAP4 is localized to the principal piece under basal conditions and in the principal piece, mid-piece and the post-acrosomal region under PMA stimulation. At least 10 images from each treatment were collected and a representative image is shown. *Scale bars* indicate 5 μm.

In order to follow the fate of proAKAP4 after phosphorylation by ERK1/2, we first followed the cellular localization of proAKAP4 in transfected HEK293T cells. The cells were transfected with tGFP-proAKAP4 or tGFP-proAKAP4-T265A, point mutation in the ERK1/2 phosphorylation site, and then were pretreated with or without the MEK/ERK inhibitor U0126 for 20 min. Thereafter PMA was added for 5–30 min (Fig. 7A). PMA induced translocation of proAKAP4 to the Golgi, as evident by the colocalization with the Golgi marker, GRASP65 (Fig. 7B). Treatment with U0126 prior to the stimulation with PMA prevented proAKAP4 translocation to the Golgi. In addition, proAKAP4-T265A did not translocate to the Golgi by PMA. The results reveal that ERK1/2 phosphorylation is important for the cellular distribution of proAKAP4.

### AKAP4 translocates to the mid-piece and the postacrosomal region following PMA treatment in human spermatozoa

We then assessed whether AKAP4 also translocates in PMA-treated spermatozoa. To that aim we followed the localization of AKAP4 in capacitated spermatozoa under basal and PMA-activated cells. Indirect immunofluorescence microcopy showed that AKAP4 is localized to the principal piece under basal conditions and in the principal piece, mid-piece and the postacrosomal region under PMA stimulation (Fig. 7C). In order to validate the specificity of the AKAP4 antibody, we ran a full-uncut WB for human sperm lysate (Supplementary Figure-S1). As can be seen, the main bands are proAKAP4 and AKAP4. The BSA band is due to the capacitation medium, which contains 0.3% BSA.

### cAMP inhibits PMA-induced ERK1/2 activation and acrosome reaction

**Figure 8:**
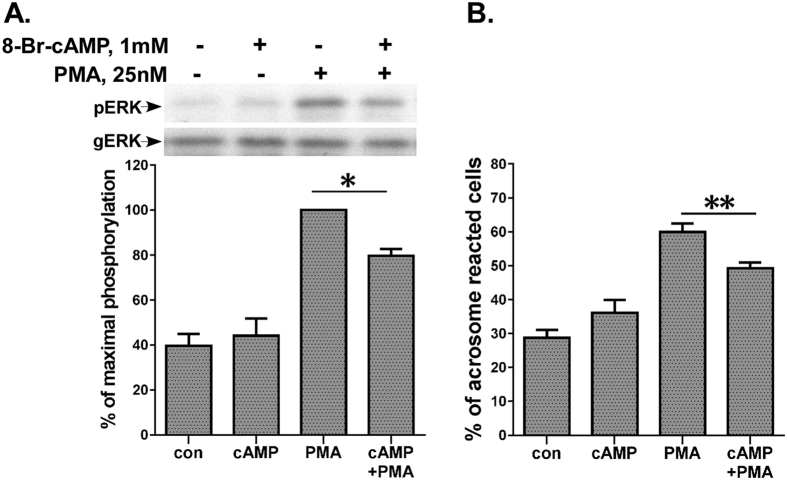
cAMP attenuates PMA-stimulated ERK1/2 activation and acrosome reaction. (**A**) Human sperm were preincubated in capacitation medium for 3 h. 8-Br-cAMP (1 mM) was added for the last 10 min of the preincubation, and PMA (25 nM) was added for another hour. Sperm lysates were analyzed for ERK2 activity by western blotting as above. A representative blot is shown and *bars* are mean ± SEM from three experiments. (**B**) Human sperm were preincubated in capacitation medium for 3 h. 8-Br-cAMP (1 mM) was added for the last 10 min of the preincubation, and PMA (25 nM) was added for another hour. The percentage of acrosome-reacted cells was determined using FITC-conjugated Pisum sativum agglutinin (PSA-FITC) as described in methods. The data represent the mean ± SEM of duplicates from 3 experiments. Means designated by * and ** are significantly different (p < 0.05 and p < 0.01), respectively.

We then investigated the biological significance of the negative crosstalk between the cAMP/PKA/AKAP4 and the PKC/ERK1/2 pathways. First, we examined the effect of cAMP upon PMA-stimulated motility. In noncapacitated cells as above (Fig. 2) cAMP had no effect on PMA stimulated progressive and hyperactivated motility (data not shown). We assume that the reduction in ERK1/2 observed in Fig. 2 (25%), was not sufficient to reduce motility. On the other hand, the use of the MEK-ERK1/2 inhibitor U0126, abolished PMA-induced ERK1/2 activity and PMA-induced motility in our previous study^26^. We then examined the effect of cAMP upon PMA-stimulated ERK1/2 activity in capacitated spermatozoa. Indeed, cAMP inhibited PMA-induced ERK1/2 activation, similar to what we saw in noncapacitated spermatozoa (Fig. 8A). We then examined the effect of cAMP upon PMA-stimulated capacitation and acrosome reaction. The capacitation state of the sperm was confirmed after the 3 h incubation in capacitation medium by examining the ability of the sperm to undergo the acrosome reaction^43^. cAMP had a significant inhibitory effect on PMA-stimulated acrosome reaction (Fig. 8B), in parallel to its inhibitory effect on ERK1/2 activation (Fig. 8A). Hence, the negative crosstalk between the cAMP/PKA/AKAP4 and the PKC/ERK1/2 pathways is involved in the regulation of capacitation and acrosome reaction.

## Discussion

AKAPs are a wide family of proteins, known to bind multiple signaling molecules that were originally co-purified with PKA-R, but many of them have been shown to anchor PKC^16^, protein phosphatase 1 (PP1), glycogen synthase kinase 3 (GSK3), protein kinase D (PKD), phosphodiesterase (PDE) and others^44^,^45^. Hence, AKAPs integrate intracellular signals by sequestering PKA with other kinases and signaling molecules.

AKAPs play an important role in spermatozoa compartmentalization by enabling the targeting of proteins and other signaling molecules to specific intracellular sites. A major protein component of the spermatozoa fibrous sheath is AKAP4^46^ that comprises ~50% of the sheath and is known to be crucial to spermatozoa motility^23^,^47^,^48^. It is therefore not surprising that AKAP4 is expressed in a variety of mammalian species and is highly conserved^49^. Nevertheless, the mechanism by which AKAP4 regulates and coordinates spermatozoa functions is not well understood. In this study we report that AKAP4 is an ERK1/2 substrate and serves as a regulator between the cAMP/PKA/AKAP4 and the PKC/ERK1/2 pathways in human spermatozoa.

AKAP4 undergoes serine and tyrosine phosphorylation during capacitation^22^, but the nature of the kinases involved are still not known. Here we show that proAKAP4 undergoes *in vitro* phosphorylation by ERK2, and mapped the phosphorylation site at Thr265. Indeed, Thr265 is a consensus sequence for ERK1/2 phosphorylation (PRTP). Furthermore, we could demonstrate the phosphorylation of proAKAP4 by MAPK *in vivo* in PMA-stimulated spermatozoa. Inhibition of the phosphorylation by the MEK inhibitor U0126 and the ERK1/2 inhibitor, GDC-0994, which prevents ERK1/2 activity through steric effects, without preventing MEK phosphorylation of ERK1/2, implicated ERK1/2 as the MAPK involved in proAKAP4 phosphorylation *in vivo.* It is well-recognized that phosphorylation of proteins on tyrosine residues is a marker of sperm capacitation^3^ and appears to be cAMP/PKA-dependent, but exactly how cAMP/PKA then stimulates tyrosine kinases and protein tyrosine phosphorylation in the sperm is unclear. One of the suggested mechanism is that the interaction may involve phosphorylation of serine or threonine residues by PKA that primes the proteins for phosphorylation on tyrosine residues^28^. Interestingly, other studies have shown that inhibition of ERK1/2 reduced the capacitation dependent induction of tyrosine-phosphorylated fibrous sheath proteins in human (80 kDa) and boar (97 kDa) spermatozoa, even suggesting that the human protein (80 kDa) could be an AKAP containing phosphorylation motif by ERK1/2^39^,^50^. Another study showed that the tyrosine kinase activity during mouse sperm capacitation is mediated also by ERK1/2^51^ and lastly, the ability of AKAP4 to bind to the regulatory subunit of PKA (RII) may be regulated by its phosphorylation by ERK1/2 as described for the AKAP RSP3H^52^.

When we examined the effect of cAMP, a vital molecule in sperm physiology^3^,^30^,^53^, we found that it had no effect on sperm ERK1/2 activity as compared to the robust effect of PMA. However, we did find that cAMP via PKA had a negative effect on PMA-induced activation of ERK1/2 (mainly ERK2). It is also known that AKAP12/Gravin binds PKC and downregulates the activation of ERK1/2 by PMA^16^. Others have shown that PKA activates ERK1/2 through Ras activation of either B-Raf or Raf-1^54^,^55^,^56^. In addition, cAMP activates ERK1/2 via B-Raf and Rap1 in PC12 cells and not by the classical pathway of Ras/Raf-1^57^. If B-Raf has a low expression level in the cells, the PKA pathway leads to inhibition of the ERK1/2 pathway by inhibition of Raf-1 via phosphorylation of Ser43 and Ser259, known as inhibitory sites in Raf-1^32^,^58^,^59^,^60^,^61^. Human spermatozoa are known to express Raf-1 but not A and B-Raf^40^ and therefore the negative effect of cAMP on ERK1/2 activity can be mediated by inhibition of Raf-1. Therefore the effect of cAMP/PKA on the PKC/Raf-1/MEK/ERK1/2 pathway is cell-context dependent^31^,^62^.

We were then interested to know if proAKAP4 participates in the negative regulation of PMA-induced ERK1/2 activation by cAMP. Since ejaculated sperm cannot be transfected we resorted to HEK293T cells, in which cAMP had no effect on basal ERK1/2 levels as in sperm. Indeed, cAMP can decrease PMA-induced ERK1/2 activation only in HEK293T cells expressing proAKAP4. Further support that proAKAP4 participates in the cAMP inhibition of PMA-induced ERK1/2 activation was derived by the use of St-HT31. The peptide drug disrupts PKARII anchoring to AKAPs^41^. The stearated peptide, St-HT31 abolished the inhibitory effect of cAMP on PMA induced ERK1/2 activation in human spermatozoa, indicating that an AKAP is involved in cAMP inhibition of PMA to ERK1/2 signaling. Still we wanted to implicate proAKAP4 in this process and we therefore resorted to HEK293T cells expressing proAKAP4. Indeed, St-HT31 abolished the inhibitory effect of 8-Br-cAMP on PMA induced ERK1/2 activation. In both models St-HT31-P (a non-active peptide) had no effect. In addition, we have also found interaction between proAKAP4 and Raf-1 in human sperm, and therefore the negative effect of cAMP/PKA on ERK1/2 is possibly mediated by inhibition of Raf-1. The collective data supports the view that proAKAP4 is involved in cAMP inhibition of PMA-induced ERK1/2 activation in human spermatozoa. It was shown that AKAP-Lbc augments signal transmission through the ERK1/2 cascade by directing PKA phosphorylation of substrates^17^,^63^. In contrast, SSeCKS/Gravin/AKAP12 inhibits the PKC-Raf/MEK/ERK pathway by disengagement of Src from its effectors Raf/MEK/ERK^16^, or by inhibition of PKC-induced activation of Raf probably by scaffolding of PKC by SSeCKS. Thus, various AKAPs can affect the PKC-ERK1/2-MAPK cascades in a cell-context and signaling-dependent manner.

Localization of AKAP4 in human spermatozoa may point to its role in sperm functions. We first focused our attention to transfected HEK293T cells, since mature sperm can’t be transfected. PMA treatment relocated proAKAP4 from the cytosol to the Golgi, as evident by colocalization with the Golgi marker, GRASP65. The effect of PMA was blocked by the MEK inhibitor, U0126 indicating that ERK1/2 was most likely involved by phosphorylation of proAKAP4. This was confirmed by showing that proAKAP4-T265A did not translocate to the Golgi by PMA. Importantly, during spermatogenesis the Golgi forms the acrosome vesicle^64^ and therefore the translocation of proAKAP4 in HEK293T provided an insight to our next observations in AKAP4 localization in spermatozoa. We then followed the localization of AKAP4 in capacitated spermatozoa under basal and in PMA-activated cells. We found that AKAP4 is expressed in the principal piece under basal conditions and in the principal piece, mid-piece and the postacrosomal region under PMA stimulation. In addition, porcine proAKAP4 was localized by immunofluorescence and subcellular fractionation to the periacrosomal membranes^65^. Since PKC-Raf-1-ERK1/2 (mainly ERK2) are also present in the sperm tail^24^,^26^,^40^, it is an ideal site to phosphorylate AKAP4 and implicate it in sperm functions.

We then investigated the biological significance of the negative crosstalk between the cAMP/PKA/AKAP4 and the PKC/ERK1/2 pathways. Although both cAMP and PMA stimulated forward motility on their own, their combination had no effect on motility (data not show). We hypothesize that the reduction in ERK1/2 observed in Fig. 2 (25%), was not sufficient to reduce motility. On the other hand, the use of the MEK-ERK1/2 inhibitor U0126, abolished PMA-induced ERK1/2 activity and PMA-induced motility in our previous study^26^. The occurrence of sperm capacitation is a prerequisite for a normal acrosome reaction. The capacitation state of the sperm is confirmed after 3 h incubation in capacitation medium by examining the ability of the sperm to undergo the acrosome reaction^43^. Therefore, capacitation and acrosome reaction can be regarded as continues process. We therefore used capacitated sperm and found that addition of cAMP reduced PMA-induced ERK1/2 activation and acrosome reaction (Fig. 8). Moreover the link between ERK1/2 activation and acrosome reaction was already established when we showed that the use of the MEK-ERK1/2 inhibitor, U0126 markedly inhibited PMA-induced acrosome reaction^26^. We therefore propose that the negative crosstalk between the cAMP/PKA/AKAP4 and the PKC/ERK1/2 pathways is to regulate capacitation and acrosome reaction.

The cAMP/PKA and the PKC/ERK1/2 pathways play major roles in sperm biology^3^,^26^,^27^,^30^,^38^,^39^, but relatively little is known about their crosstalk and the signaling molecules that can co-regulate them in particular in human spermatozoa. Here we demonstrate that AKAP4 is such a molecule that can serve as a regulator of the cAMP/PKA and the PKC/ERK1/2 pathways in human spermatozoa to regulate capacitation and acrosome reaction.

## Methods

### Plasmid constructs antibodies and reagents

Media, sera and antibiotics for cell cultures were obtained from Biological Industries (Kibbutz Beit Ha’Emek, Israel). PMA, U0126, IBMX and 8-Br-cAMP were obtained from Sigma (Rehovot, Israel). GDC-0994 was obtained from Selleckchem (Houston, TX, USA). Myristoylated PKI 14–22 amide (PKI) was obtained from Tocris Bioscience (Bristol, United Kingdom). St-HT31 and St-HT31-P were obtained from promega (Madison, Wisconsin, USA). jetPRIME Transfection reagent was obtained from polyplus transfection (Illkirch, France). Proteins A/G agarose beads were obtained from Santa Cruz Biotechnology, Inc. (Santa Cruz, CA). [γ-^32^P]ATP was obtained from PerkinElmer (MA, USA). Recombinant ERK2 was obtained from New England Biolabs (Ipswich, MA, USA). Mouse monoclonal anti-doubly phosphorylated (DP)-ERK1/2 antibodies and rabbit polyclonal antibodies to general ERK and rabbit anti-AKAP4 were obtained from Sigma-Aldrich (Rehovot, Israel). Rabbit anti-turboGFP was obtained from Evrogen (Moscow, Russia). Mouse monoclonal anti-phospho-Ser/Thr-Pro (MPM2) antibody was from Upstate (Lake Placid, NY, USA). Secondary horseradish peroxidase-conjugated goat anti mouse or goat anti rabbit antibodies were purchased from Jackson ImmunoResearch Laboratories (West Grove, PA). Secondary anti-rabbit Alexa Fluor™ 488-labeled antibody was obtained from Thermo Fisher Scientific (Waltham, MA USA). Anti Raf-1 antibody was from Merck Millipore (Darmstadt, Germany). tGFP-tagged ORF clone of human AKAP4 transcript variant 1 (NM_003886.2) was obtained from Origene (Rockville, MD). GRASP65-RFP construct was kindly provided by Dr. K. Hirschberg (Tel-Aviv University, Israel).

### Preparation of human spermatozoa

Human semen was obtained from healthy donors with normal sperm density, motility, and morphology according to World Health Organization guidelines^66^. All the experimental protocols were approved and performed in accordance with relevant guidelines and regulations of the Tel Aviv University Helsinki Committee, and informed consent was obtained from all the subjects. The human semen was liquefied for 60 min at 37 °C. Sperm was washed twice with Ham’s F-10 medium containing bovine serum albumin (BSA, 0.3%) and incubated with the medium for the indicate time or for 3 h for capacitation.

### Assessment of sperm motility

Progressive flagellar motility was determined by using Computer-aided Sperm Analysis (CASA) (Sperm Analysis System version 12-IVOS, Hamilton Thorne Biosciences, Beverly, MA).

### Assessment of sperm capacitation and acrosome reaction

Washed cells were capacitated for 3 h at 37 °C and 5% CO_2_ in F-10 medium supplemented with BSA (3 mg/ml), the capacitation medium. The capacitation state of the sperm was confirmed after the 3 h incubation in by examining the ability of the sperm to undergo the acrosome reaction, which was induced by the addition of 25 nM PMA for 1 h. At the end of incubation an aliquot of the sperm was spread on microscope slides and allowed to air-dry. The sperm were then permeabilized by methanol for 15 min at room temperature, washed once with 25 mM Tris-buffered saline, pH 7.6, for 5 min and twice with H_2_O at 5-min intervals, air-dried, and then incubated with FITC-P. sativum agglutinin (60 μg/ml) for 1 h, washed twice with H_2_O at 5-min intervals, and mounted with FluoroGuard Antifade (Bio-Rad). The percentage of acrosome-reacted sperm was determined microscopically on air-dried sperm smears using FITC-conjugated Pisum sativum agglutinin, which is a fluorescent lectin capable of binding to the acrosomal content. For each experiment, at least 150 cells per slide in duplicates were evaluated. Cells with green staining over the acrosomal cap were considered acrosome-intact; those with equatorial green staining or no staining were considered acrosome-reacted.

### Activation of MAPK cascades

Capacitated and noncapacitated human spermatozoa were prepared as above and stimulated with the proper drug and cell extract was used for Western blotting. After stimulation, the cells were precipitated by centrifugation at 15,000 × *g* for 5 min at 4 °C, and washed once more, and the resulted pellets were resuspended in a minimal volume of lysis buffer (50 μl per 3 × 10^7^ cells) made of 50 mM Tris-HCl, pH 8.0, 2 mM EGTA, 20 mM NaCl, 1 mM sodium orthovanadate, 25 mM β-glycerophosphate, 100 nM okadaic acid, 1.5% Nonidet P-40, 1 mM benzamidine, 10 μg/ml aprotinin, 10 μg/ml leupeptin, 1 mM phenylmethylsulfonyl fluoride, and 2 mM dithiothreitol. The suspensions were incubated on ice for 30 min and centrifuged (15,000 × *g*, 15 min, 4 °C). The supernatants were collected and mixed with Laemmli loading buffer and aliquots from each sample were separated on 10% SDS-PAGE followed by Western blotting with the proper antibody. The blots were developed with horseradish peroxidase-conjugated anti-mouse or anti-rabbit Fab antibodies. The blots were autoradiographed on Fuji Super RX films and the phosphorylation was quantified by ImageJ (NIH, Bethesda, MD, USA). Each band from the anti-phospho-MAPK was normalized to the corresponding band from the anti-general MAPK antibodies blot for even loading.

### Identification of MAPK substrates

Human spermatozoa were washed and stimulated with 100 nM PMA for 15 min. Cells were lysed and resolved on 10% SDS-PAGE and western-blotted with anti-MPM2, a phospho-specific anti-MAPK substrate antibody^21^. The original gel was used to derive another substrate ARHGAP6 and was therefore presented previously^21^. Major PMA-stimulated bands were subjected to MALDI-TOF mass spectrometry.

### Immunoprecipitation

Human spermatozoa were prepared as above and stimulated with the proper drug and cell extract was used for immunoprecipitation. After stimulation, the cells were precipitated by centrifugation at 15,000 × g for 5 min at 4 °C, and washed once more with ice-cold PBS and overlaid with 0.5 ml of lysis buffer (as above) on ice for 30 min, then sonicated three times for 30 sec each time, followed by centrifugation (15,000 × g, 15 min, 4 °C). The supernatants were collected for further experiment. For immunoprecipitation, cell lysates were mixed with the appropriate antibody (2 μg) and incubated and rotated over night at 4 °C. Then, protein A/G Agarose beads were added to the cell lysates and rotated at 4 C for 3 hours. The immunocomplexes were washed three times with the lysis buffer and heated to 100 °C in Laemmli loading buffer. The supernatants were collected and separated on 10% SDS PAGE, followed by Western blotting. For all experiments control immunoprecipitations were performed, where the antibody was omitted and only A/G beads were added to the cell lysate.

### Site-directed mutagenesis

10 ng of tGFP-proAKAP4 were added to 125 ng of each primer, dNTP mix and 1 μl of PfuUltra DNA polymerase (Ageilent technologies), and DDW to 50 μl total volume. Cycling parameters were according to the primers Tm and manufacturer’s instructions. Primers were: forward: gaa tca gtc ccc gag ctc ctg cga gca ag reverse: ctt gct cgc agg agc tcg ggg act gat tc. Following, parental methylated DNA was digested with 1 μl DpnI for 1 hour. DNA was transformed by heat shock into XL1b competent cells. 50 ng DNA was added to the cells, left for 30 minutes in ice, then heat-shocked for 45 seconds at 42 °C, left in ice for 2 minutes and incubated with fresh LB medium for 1 hour in 37 °C. The cells were then plated on agar that contained the proper selection antibiotics. The mutants were sequenced to verify the mutations.

### Deletion mutations

Primers were phosphorylated by polynucleotide kinase (New England Bioloabs) according to manufacturer’s manual. The primers were added to the template, dNTPs, PfuUltra DNA polymerase (Ageilent technologies), and DDW as described in the previous section and thermo-cycled according to manufacturer’s instructions. Following, parental methylated DNA was digested with 1 μl DpnI for 1 hour. The PCR products were then ligated with T4 ligase (New England Bioloabs) for 16 hours at 4 °C, transformed to XL1b cells, grown with the proper antibiotics and the DNA was sequenced to verify the mutation. Primers were: forward: gcg agc aag att gct tct gaa atg, reverse: cag tgc atg ttg gaa acc caa g.

### Cell culture and transfection

LβT2 gonadotrope cells and HEK293T cells were grown in DMEM supplemented with 10% FCS, streptomycin (100 μg/ml), penicillin (100 units/ml) and 5% glutamine. Cells were maintained in humidified atmosphere of 5% CO_2_ and at 37 °C. HEK293T Cells were plated in 12 wells plates 24 h prior to transfection and were transfected with 1 μg of plasmid DNA. Approximately 24 h after transfection, cells were serum starved (0.1% FCS) for 16 h and then stimulants were added. After that the cells were washed twice with ice-cold PBS and treated with lysis buffer for 15 min at 4 °C. Cells were harvested and were centrifuged (15,000 g, 15 min, 4 °C). Cell supernatants were mixed with sample buffer, and ran on 10% SDS-PAGE followed by Western blotting and incubation of the membrane with the appropriate antibodies.

### *In Vitro* Phosphorylation

HEK293T Cells were plated in 100-mm plates 24 h prior to transfection with tGFP-proAKAP4 and it mutants followed by transfection with 10 μg of plasmid DNA. 48 h later the cells were lysed with 100 mM NaCl, 20 mM Tris-HCl, pH 7.4, 0.5 mM EDTA, 15% glycerol, 0.2% Triton X-100, 1 mM phenylmethylsulfonyl fluoride, and protease inhibitors mixture. The lysate was subjected to immunoprecipitation with an anti-turbo-green fluorescent protein antibody conjugated to protein A/G PLUS-agarose beads by gentle shaking overnight in 4 °C. The beads were washed once in RIPA buffer, followed by three washes with LiCl 0.5 M Tris 0.1 M pH = 7.4 and another wash in RIPA buffer in order to remove proteins that bound non-specifically to the beads and might have co-precipitated with tGFP-proAKAP4 or its mutants. Samples were frozen in −20 °C until their subjection to *in vitro* phosphorylation. The immunoprecipitated tagged protein attached to beads (0.5–1 μg per reaction) was mixed with purified ERK2. The buffer reaction mix contained (3×) (75 mM β-glycerophosphate, 1.5 mM dithiothreitol, 3.8 mM EGTA, 0.15 mM orthovanadate, 30 mM MgCl_2_, 30 mM calmidozolium, 0.3 mM ATP). 100 μM [γ-^32^P] ATP (4000 cpm/pmol) was added to the reaction and incubated for 10 min at 30 °C. The reaction was terminated by adding sample buffer, and the phosphorylated proteins were resolved on 10% SDS-PAGE, transferred to nitrocellulose membrane (1.5 h at 300 mA) and autoradiographed.

### Fluorescent imaging

HEK293T cells were plated on 18 mm cover slips and transfected using the calcium phosphate method with 1 μg tGFP-proAKAP4, tGFP-proAKAP4-T265A or GRASP5. 24 h after transfection, cells were serum starved (16 h, 0.1% FCS) and were pretreated with U0126 for 20 min and then PMA was added for various time points. Subsequently, the cells were washed with PBS, fixed in 4% paraformaldehyde (PFA) in PBS and then probed with DAPI. Cover slips were placed on glass slides with Vectashield mounting medium. Confocal fluorescent images were collected with a 63x magnification by Zeiss LSM-META confocal microscope.

### Immunofluorescence

Sperm were washed and smeared on polylysine precovered slides and then allowed to air-dry. Cells were washed with PBS, permeabilized with Triton X-100 0.5% buffered in PBS. Nonspecific binding was blocked with 3% BSA buffered in PBS. Cells were probed first with monoclonal anti-AKAP4 (1:75), washed three times with PBS, and then probed with a secondary anti-rabbit Alexa Fluor™ 488-labeled antibody. Slides were viewed with Zeiss LSM-META confocal microscope.

### Data Analysis

Results from two or more experiments were expressed as mean ± SEM. Where appropriate, data were subjected to statistical analysis by Student’s t test. Values of P < 0.05 were considered statistically significant.

## Additional Information

**How to cite this article**: Rahamim-Ben Navi, L. *et al*. A-Kinase Anchoring Protein 4 (AKAP4) is an ERK1/2 substrate and a switch molecule between cAMP/PKA and PKC/ERK1/2 in human spermatozoa. *Sci. Rep.* **6**, 37922; doi: 10.1038/srep37922 (2016).

**Publisher's note:** Springer Nature remains neutral with regard to jurisdictional claims in published maps and institutional affiliations.

## Supplementary Material

Supplementary Information
