# Supplementary material for: A-Kinase Anchoring Protein 4 (AKAP4) is an ERK1/2 substrate and a switch molecule between cAMP/PKA and PKC/ERK1/2 in human spermatozoa
Source: Sci Rep. 2016 Nov 30;6:37922. doi: 10.1038/srep37922 (PMC5128789; doi:10.1038/srep37922)
Supplement: Supplementary Information [file srep37922-s1.pdf]

A-Kinase Anchoring Protein 4 (AKAP4) is an ERK1/2 substrate and a switch molecule between cAMP/PKA and PKC/ERK1/2 in human spermatozoa

Liat Rahamim Ben-Navi[a,+], Tal Almog[a,+], Zhong Yao[b], Rony Seger[b], Zvi Naor[a,*]

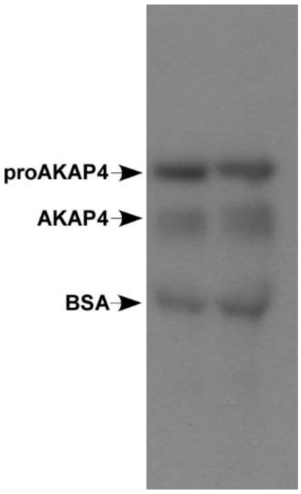

Figure S1. Specificity of the AKAP4 antibody. Human sperm were preincubated in capacitation medium. Cells were then lysed and analyzed for AKAP4 by western blotting using an antibody for AKAP4.
